# Multi-Target Neuroprotective Effects of Flavonoid-Rich *Ficus benjamina* L. Leaf Extracts: Mitochondrial Modulation, Antioxidant Defense, and Retinal Ganglion Cell Survival In Vivo

**DOI:** 10.3390/ijms262311746

**Published:** 2025-12-04

**Authors:** Arik Dahan, Moria Oz, Ludmila Yarmolinsky, Alon Zahavi, Nitza Goldenberg-Cohen, Boris Khalfin, Shimon Ben-Shabat, Bat Chen R. Lubin

**Affiliations:** 1 Department of Clinical Pharmacology, School of Pharmacy, Faculty of Health Sciences, Ben Gurion University of the Negev, Beer-Sheva 8410501, Israel; yludmila@post.bgu.ac.il (L.Y.); boriskh83@gmail.com (B.K.); sbs@bgu.ac.il (S.B.-S.); 2 Department of Molecular Biology, Faculty of Natural Sciences, Ariel University, Ariel 40700, Israel; moriaoz8@gmail.com; 3 Ophthalmology Department and Eye Research Laboratory, Felsenstein Medical Research Center, Rabin Medical Center, Petach Tikva 4941492, Israel; alonzahavi@gmail.com; 4 Gray Faculty of Medical and Health Sciences, Tel Aviv University, Tel Aviv 6997801, Israel; 5 Department of Ophthalmology, Bnai-Zion Medical Center, Haifa 3339419, Israel; ncohen1@technion.ac.il; 6 Krieger Eye Research Laboratory, Ruth and Bruce Faculty of Medicine, Technion—Institute of Technology, Haifa 3200003, Israel; 7 Department of Chemical Engineering, Biotechnology and Materials, Ariel University, Ariel 40700, Israel; 8 Agriculture and Oenology Department, Eastern Regional R&D Center, Ariel 40700, Israel

**Keywords:** *Ficus benjamina*, flavonoids, neuroprotection, oxidative stress, traditional Chinese medicine, ethnopharmacology

## Abstract

Oxidative-stress-induced neuronal injury is a major contributor to neurodegenerative diseases, underscoring the need for novel neuroprotective strategies. Natural products with antioxidant and mitochondrial-stabilizing properties are increasingly recognized as promising multi-target therapeutics. *Ficus benjamina* L., a member of the Moraceae family, is rich in flavonoids and traditionally used in Asian ethnomedicine for wound healing, inflammation, and weakness, with related Ficus species documented in the *Bencao Gangmu* (*Compendium of Materia Medica*) for circulation and detoxification disorders. However, its neuroprotective potential has not been systematically evaluated. In this study, we explored the neuroprotective potential of a flavonoid-enriched 80% methanolic leaf extract of *F. benjamina* by evaluating its capacity to mitigate oxidative stress in neuronal cells and a murine optic nerve crush (ONC) injury model. We observed in SH-SY5Y cells that cell viability was preserved after pre-treatment using the extract, mitochondrial respiration and the membrane potential were maintained, and gene expression was modulated by upregulation of *BCL-2* (B-cell lymphoma 2), *BCL-xL* (*B-cell lymphoma-extra large*), X) *SOD2* (Superoxide Dismutase), *CAT* (Catalase), and *BDNF* (Brain-Derived Neurotrophic Factor). Intravitreal delivery of the extract in vivo resulted in a marked increase in the survival of retinal ganglion cells following ONC injury. Caffeic acid, quercetin-3-O-rutinoside, and kaempferol-3-O-rutinoside were identified as major constituents in phytochemical profiling. These results indicate that *F. benjamina* exerts multi-target neuroprotective actions, mediated via mitochondrial regulation, enhancement of antioxidation defenses, and modulation of apoptotic pathways. The findings also substantiate the contemporary pharmacological relevance underscoring the ethnomedicinal use of *Ficus* species and highlight the potential of *F. benjamina* as a promising candidate for developing integrative therapeutic approaches to target neurodegenerative diseases driven by oxidative stress.

## 1. Introduction

Stroke is a leading cause of both death and disability worldwide [1]. Ischemic stroke accounts for approximately 87% of all stroke cases and is primarily caused by occlusion of cerebral blood vessels, leading to impaired cerebral perfusion [2]. In addition to the risk of death, stroke frequently results in profound and persistent neurological disabilities, significantly impacting the elderly population more [3]. Beyond cerebral ischemia, oxidative-stress-mediated injury affects CNS (central nerve system) neurons throughout the nervous system, including retinal ganglion cells in conditions such as ischemic optic neuropathy, traumatic injury, and glaucoma, which share pathophysiological similarities with stroke [4,5]. Most neuronal injuries that happen after a stroke are caused by complicated biochemical reactions, with oxidative stress being the most prominent [6]. In the last three decades, an increasing number of basic science studies with emphasis on the pathophysiology and mechanisms of stroke have demonstrated that neuroprotection is a promising strategy for stroke treatment [7].

Oxidative stress is characterized by the excessive generation of reactive oxygen species (ROS), including hydrogen peroxide (H_2_O_2_), which causes cellular damage via various mechanisms, including mitochondrial dysfunction, dysregulation of gene expression, and the induction of apoptosis [8]. These processes have a significant effect on the expression of vital neuronal markers such as Brain-Derived Neurotrophic Factor (BDNF) and Aromatic L-amino Acid Decarboxylase (AADC), as well as the activity of intrinsic antioxidant enzymes like Superoxide Dismutase (SOD2) and Catalase (CAT) [9,10,11].

Neuroprotection remains a critical component in the management of ischemic neuronal injury, motivating strategies by developing approaches that limit secondary injury from oxidative stress, excitotoxicity, and apoptosis [12]. Pharmacological neuroprotective strategies are not successful and often associated with numerous side effects [13]. Conventional approaches such as calcium channel blockers, glutamate receptor antagonists, and antioxidants or free radical scavengers are primarily intended to provide pharmacological neuroprotection. However, there are still not many treatment options, which has led to a lot of interest in natural compounds from medicinal plants that contain a wide range of bioactive molecules that can target multiple pathways at once [14,15,16]. Novel therapeutic approaches, incorporating drug candidates explicitly intended to target multiple neural and biochemical processes, may propose more accurate treatments [17,18].

Herbal extracts are being progressively acknowledged for their ability to regulate multiple protein targets simultaneously, thus providing an advantage in addressing diverse neural pathways implicated in neurodegeneration. Extracts contain a mix of different bioactive compounds, like polyphenols, phenolic acids, flavonoids, and alcohol derivatives. These compounds may work together to make the desired therapeutic effect more likely [19,20].

The search for neuroprotective compounds that originate from natural sources has attracted considerable interest because of their potential therapeutic benefits and reduced side effects [21,22].

*Ficus benjamina*, commonly referred to as the weeping fig, has shown favorable pharmacological properties, such as antioxidant and anti-inflammatory activities, positioning it as a potential candidate for novel therapeutic development.

Traditional medicine has used the leaves, bark, and latex of *F. benjamina* to treat wounds, skin conditions, gastrointestinal disorders, and respiratory issues because of their antimicrobial and wound-healing properties [23,24]. Researchers have investigated a wide range of bioactivities in the Ficus species, such as their ability to fight cancer, bacteria, viruses, inflammation, and free radicals [25,26,27]. Even though many Ficus species have been studied for their biological activities, the specific neuroprotective abilities of *F. benjamina* remain fundamentally unexplored.

In this work, we aimed to examine the potential neuroprotective properties of *F. benjamina* plant extracts and their constituents that are known in the ethno-botanic literature but have not yet been tested using scientific tools for their neuroprotective potential. In Traditional Chinese Medicine (TCM), Ficus species have long been employed for their effects on circulation, inflammation, and tissue repair. The *Bencao Gangmu* (*Compendium of Materia Medica*) has documented *Ficus carica* and *Ficus hispida*, for example, as plants that have traditionally been used to improve blood flow, remove toxins, and heal wounds [28,29].

*F. benjamina* is not traditionally included in classical materia medica; however, its closely related species are used in TCM prescriptions for conditions associated with “heat-toxins,” inflammation, and weakness [25,28,30]. Recent ethnopharmacological studies across Asia show the use of *F. benjamina* in the treatment of ailments such as cough, fever, and neurological weakness, aligning with the TCM principle of restoring balance and qi circulation (the movement of vital energy (qi) through the body) [31]. Yet, it fails to provide experimental validation of its effectiveness in neurological or visual disorders, and mechanistic neuroprotective studies are yet to be carried out.

## 2. Results

### 2.1. Evaluation of the Neuroprotective Effects of Plant Extracts on H_2_O_2_-Treated SH-SY5Y Cells

**Figure 1:**
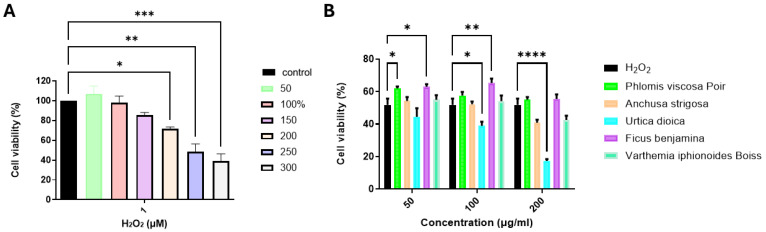
Effect of 5 different alcoholic plant extracts against H_2_O_2_-induced cell damage. (**A**) SH-SY5Y cell viability treated with different concentrations of H_2_O_2_ for 12 h using an XTT assay. (**B**) SH-SY5Y cells were pre-treated with different concentrations of 5 different alcoholic plant extracts for 6 h and oxidative stress induction with 250 µM H_2_O_2_ for 12 h. * *p* < 0.05, ** *p* < 0.01; *** *p* < 0.001, **** *p* < 0.0001 compared to H_2_O_2_-treated cells.

One of the causes of neurodegenerative disease is the formation of oxidative stress in the cells [8]. Thus, initially, we incubated SH-SY5Y cells in a range of concentrations of H_2_O_2_ to induce oxidative stress, and cell viability was determined. The results showed a decrease in cell viability in a dose-dependent manner (Figure 1A). A concentration of 250 μM of H_2_O_2_ decreased cell metabolism by 50%, and this concentration was chosen for further experiments. Five different plants were evaluated for their neuroprotective effect against oxidative stress on SH-SY5Y cells *F. benjemina*, *P. viscosa*, *U. dioica*, *A. strigosa*, and *V. iphionoides*. Clear differences in cell survival rate were apparent following treatment with plant extracts in a dose-dependent manner. The neuroprotective effects of *P. viscosa Poiret* and *F. benjemina* were significant. The former had a neuroprotective effect at a concentration of 50 μg/mL, and a 62% cell survival rate was observed (*p* < 0.01). Meanwhile, *F. benjemina* demonstrated neuroprotective effects at a concentration of 50 µg/mL and 100 µg/mL with 62% and 65% cell survival, respectively (*p* < 0.01, *p* < 0.001). *U. dioica*, *A. strigose*, and *V. iphinoides* did not present a protective effect, with low cell survival (Figure 1B).

### 2.2. Evaluation of the Neuroprotective Effects of F. benjamina Fractions on H_2_O_2_-Treated SH-SY5Y Cells

**Figure 2:**
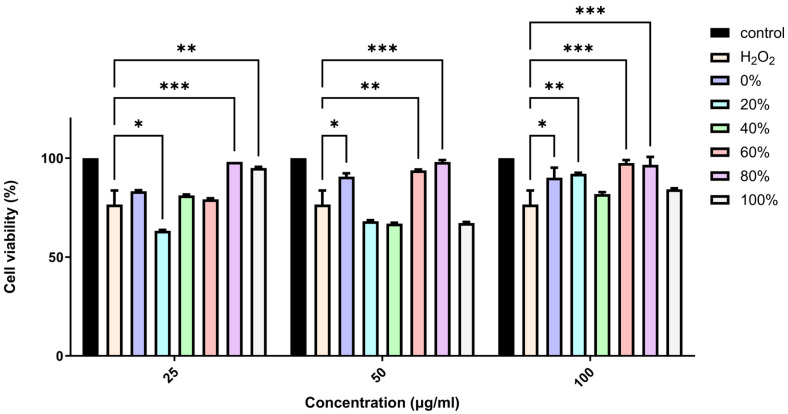
Effect of *F. benjamina* fractions against H_2_O_2_ -induced cell damage. SH-SY5Y cells were pre-treated with the indicated concentrations of the 6 ethanolic fractions of *F. benjamina* for 6 h followed by addition of 250 µM H_2_O_2_ for 12 h. * *p* < 0.05, ** *p* < 0.01, *** *p* < 0.001, compared to H_2_O_2_-treated cells.

Based on the previous results, we further investigated the neuroprotective effect of *F. benjamina* fractions. The crude ethanol extract of *F. benjamina* was fractioned. Fractions obtained were studied for their neuroprotective effect using the XTT assay. As shown in Figure 2, the fractions demonstrate a variety of effects on the cells treated with H_2_O_2_. The 60% fraction protected the cells from H_2_O_2_-induced damage in 50 and 100 µg/mL, while the 80% fraction protected the cells from H_2_O_2_-induced damage in all concentrations tested consistently.

### 2.3. Favorable Effects of F. benjamina 80% Fraction on Mitochondrial Respiration in H_2_O_2_-Treated SH-SY5Y Cells

**Figure 3:**
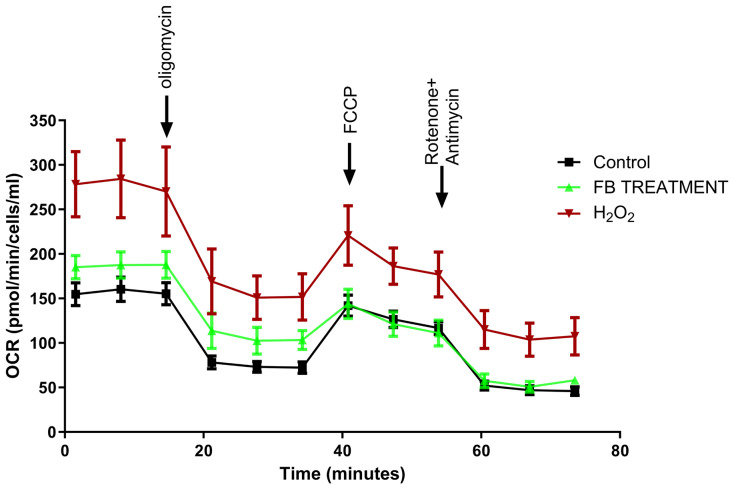
OCR over time of SH-SY5Y cells. Time course for measurement of oxygen consumption rate (OCR) is shown using Seahorse XF8 analyzer, followed by the sequential addition of oligomycin (1.5 μM), FCCP (2 μM), and rotenone (0.5 μM), combined with antimycin (0.5 μM).

Next, we determined the influence of the *F. benjamina* 80% fraction on mitochondrial respiration using the Seahorse method, a direct measurement of cellular mitochondrial function. The oxygen consumption rate (OCR) was measured at the basal level and then sequentially treated with oligomycin (Oligo), carbonyl cyanide 4-(trifluoromethoxy)phenylhydrazone (FCCP), and rotenone plus antimycin (Ant A + Rot). The OCR over time demonstrates that H_2_O_2_ induced alterations in mitochondrial respiratory activity. Pre-treatment with the 80% fraction appears to mitigate some of the extreme OCR fluctuations (Figure 3).

**Figure 4:**
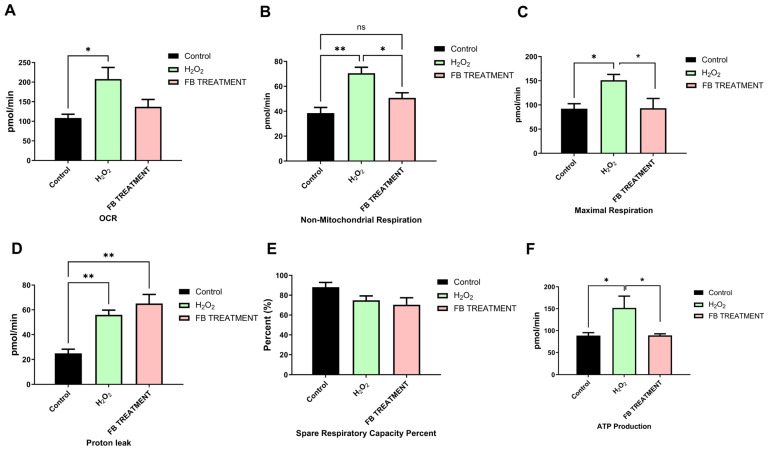
Mitochondrial respiratory parameters in SH-SY5Y pre-treated cells. (**A**) OCR: Represents overall mitochondrial respiratory activity. (**B**) Non-Mitochondrial Respiration: Indicates respiratory processes independent of mitochondrial function. (**C**) Maximal Respiration: Measures the maximum respiratory capacity of cells. (**D**) Proton Leak: Quantifies the proton flux across the mitochondrial membrane not coupled to ATP production. (**E**) Spare Respiratory Capacity Percent: Reflects the cells’ ability to increase respiratory function under stress. (**F**) ATP production: Measures mitochondrial ATP generation. Data is presented as mean ± standard deviation of the mean (SD). * *p* < 0.05, ** *p* < 0.01.

As for other mitochondria functions, it can be seen that oxidative stress induced by H_2_O_2_ increased all mitochondria functions tested. Pre-treatment with the 80% fraction reduced all parameters back to almost control levels, except for proton leak, which has no significant changes between H_2_O_2_ and 80% fraction pre-treatment. The data presented here (Figure 4) suggests that H_2_O_2_ induces significant mitochondrial stress, causing increased respiratory parameters. Pre-treatment with the 80% fraction appears to modulate these changes, generally reducing them back towards control levels, with the notable exception of proton leak, which unexpectedly increases.

### 2.4. The 80% Fraction Alleviated Induced Mitochondrial Membrane Potential Damage in SH-SY5Y Cells

**Figure 5:**
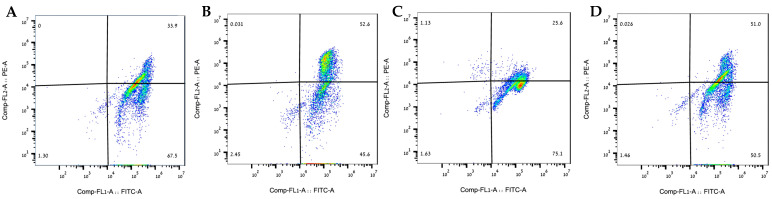
The 80% fraction alleviated the H_2_O_2_ -induced mitochondrial damage. (**A**) Control cells showing baseline mitochondrial polarization. (**B**) FCCP control, demonstrating mitochondrial membrane depolarization. (**C**) H_2_O_2_-treated cells exhibiting significant mitochondrial depolarization with reduced red fluorescence and increased green fluorescence. (**D**) Cells treated with H_2_O_2_ and 80% fraction ethanolic extract, demonstrating restoration of membrane potential similar to control condition.

JC-1 staining was used to assess mitochondrial membrane potential (ΔΨm), which is an important parameter of mitochondrial function and cellular health. The result from the JC-1 staining (Figure 5) aligns with the previous result demonstrating that the 80% fraction appears to help maintain mitochondrial membrane potential (preserving the red JC-1 fluorescence) despite the elevated proton leak observed, suggesting that the extract is promoting mitochondrial resilience and potentially protecting against oxidative-stress-induced dysfunction.

### 2.5. Multi-Target Neuroprotective Mechanisms of 80% Fraction Against Oxidative Stress

**Figure 6:**
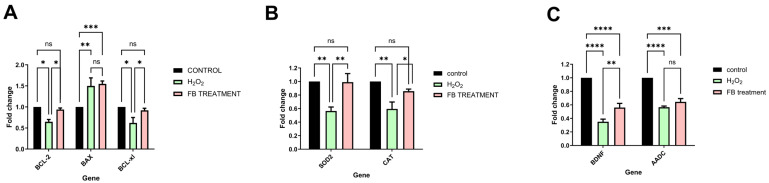
mRNA expression of neural, antioxidant, and apoptotic genes in SHSY5Y cells treated with the 80% fraction. Cells were pre-treated with the 80% fraction at 100 μg/mL for 6 h and then treated with 250 µM H_2_O_2_ for 12 h. Levels of (**A**) *BDNF* and *AADC*, (**B**) *SOD2* and *CAT*, and (**C**) *Bcl-2*, *BAX*, and *Bcl-xl* mRNAs were determined by RT-pcr. * *p* < 0.05, ** *p*< 0.01, *** *p* < 0.001, **** *p* < 0.0001.

*BCL2* (B-cell lymphoma 2) is an anti-apoptotic gene: H_2_O_2_ treatment significantly decreased BCL-2 expression (to ~0.65-fold) compared to the control (1.0), while the 80% fraction restored BCL-2 expression back to near-normal levels (~0.95-fold). *BAX* (BCL2-associated X) is a pro-apoptotic gene: Both H_2_O_2_ and 80% fraction treatments showed significantly higher *BAX* expression compared to the control. *BCL-xl* is another anti-apoptotic gene: H_2_O_2_ treatment significantly decreased *BCL-xl* expression (to ~0.6-fold) compared to the control, while the 80% fraction restored *BCL-xl* expression back to near-normal levels (~0.9-fold) (Figure 6A).

Both anti-apoptotic genes, *BCL-2* and *BCL-xl*, showed a significant decrease in treatment with H_2_O_2_ to ~0.65-fold and 0.6-fold, respectively. In contrast, pre-treatment with the 80% fraction restores gene levels almost to normal compared to the control (~0.95-fold and ~0.9-fold, respectively). In contrast, an increase was found in *BAX*, which is pro-apoptotic, while treatment with the fraction did not affect expression (Figure 6A).

*BCL2/BAX* Ratio: The *BCL2/BAX* ratio is an important indicator of cell susceptibility to apoptosis. This ratio helps determine whether a cell is likely to survive or undergo programmed cell death. Our data showed that while the H_2_O_2_ *BCL2/BAX* ratio was 0.43, pre-treatment with the 80% fraction increased the *BCL2/BAX* ratio to 0.63, suggesting partial restoration (* *p* > 0.05).

*BAX/BCL-xl* Ratio: In the H_2_O_2_ treatment, the *BAX/BCL-xl* ratio was 2.5, indicating a shift toward apoptosis. Pre-treatment with the 80% fraction reduced the *BAX/BCL-xl* ratio dramatically to 1.67 (* *p* < 0.05), indicating resistance to apoptosis. This data suggests that while the 80% fraction helps restore some balance, the cells remain somewhat shifted toward apoptosis compared to the control state.

Superoxide Dismutase (*SOD2*) and Catalase (*CAT*) are two important antioxidant enzymes. For that reason, we have evaluated their gene expression changes following 80% fraction treatment.

H_2_O_2_ treatment significantly decreased *SOD2* expression (to ~0.55-fold) compared to the control (1.0) Following pre-treatment with the 80% fraction, the *SOD2* expression levels were restored back to normal levels (~1.0-fold) (Figure 6B).

*CAT* (Catalase): H_2_O_2_ treatment significantly decreased *CAT* expression (to ~0.6-fold) compared to the control. Partial restoration was detected with 80% fraction pre-treatment (to ~0.85-fold), which is significantly higher than H_2_O_2_ but not fully back to control levels (Figure 6B).

### 2.6. BDNF (Brain-Derived Neurotrophic Factor) and AADC (Aromatic L-Amino Acid Decarboxylase)

*BDNF* (Brain-Derived Neurotrophic Factor) is a critical neurotrophic factor that supports neuronal survival, growth, and differentiation. H_2_O_2_ treatment significantly decreased *BDNF* expression (to ~0.35-fold) as compared to the control (1.0). Pre-treatment with the 80% fraction partially restored *BDNF* expression (to ~0.55-fold), which is significantly higher than with H_2_O_2_ alone, but considerably lower than the control (Figure 6C).

*AADC* (Aromatic L-amino Acid Decarboxylase) is an enzyme that helps synthesize serotonin and dopamine. H_2_O_2_ treatment significantly reduced *AADC* expression to approximately 0.55-fold compared to the control. Treatment with the 80% fraction did not significantly alter *AADC* expression compared to H_2_O_2_ alone (Figure 6C).

This data provides a more complete picture of how the 80% fraction may provide protection:

Taking together all the data, we have demonstrated that the 80% fraction provides protection against oxidative-stress-induced cellular damage through modulation of multiple molecular targets, including mitochondrial function, antioxidant defenses, and apoptotic pathways.

### 2.7. The 80% Fraction Enhances Retinal Ganglion Cell (RGCs’) Survival Following ONC

**Figure 7:**
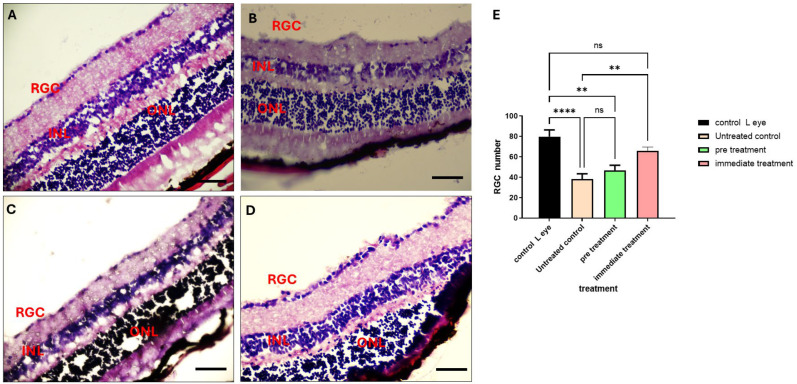
Neuroprotective effect of the 80% fraction in an ONC mouse model. (**A**) Control normal retina layer. (**B**) Untreated control. (**C**) Retinal layer after 80% fraction pre-treatment. (**D**) Retina layer after immediate 80% fraction treatment. (**E**) RGC count after both treatment timelines and compered to the control eye. Data is presented as mean ± standard deviation of the mean (SD). ** *p* < 0.01, **** *p* < 0.0001. Scale bar is 50 μm.

To further investigate the neuroprotective effect of the 80% fraction, we employed a mouse model of ONC in vivo. A total of 18 mice were used in this study (n = 6 for each treated and control group). Two injection timelines were evaluated: 1. Administration of the 80% fraction one hour prior to ONC induction. 2. Administration of the 80% fraction immediately following ONC induction. On day 21, eyes were enucleated, and retinas were carefully dissected for analysis. Histological analysis revealed that mice treated with a fraction of 80% immediately after ONC induction exhibited higher RGC counts in the injured retina compared to those treated one hour prior to ONC (Figure 7).

### 2.8. Identification of Phytochemicals

The extract of *F. benjamina* was fractionated using a stepwise methanol gradient of 0%, 20%, 40%, 60%, 80%, and 100%. All six resulting fractions were tested in vitro (Figure 2), with the 80% methanol fraction demonstrating the highest neuroprotective activity. Consequently, this fraction was selected for phytochemical analysis. The identified flavonoids include caffeic acid, quercetin 3-O-rutinoside, kaempferol 3-O-rutinoside, and kaempferol 3-O-robinobioside (Table 1). The identification of these compounds through spectroscopic techniques, including NMR and mass spectrometry, has been previously documented (Supplementary Figure S1) [26,32].

## 3. Discussion

This research underscores the neuroprotective potential of *F. benjamina*, particularly emphasizing its 80% methanolic flavonoid-enriched fraction. So far, no studies have explored how *F. benjamina* extracts affect important neuronal processes like mitochondrial function, apoptotic signaling, or neurotrophin expression under oxidative stress. The neuroprotective attributes of caffeic acid, notably its ability to mitigate neuroinflammation and interfere with ferroptosis through the modulation of the Nrf2 signaling pathway, are well documented. However, the functions of other flavonoid components within the active fraction, along with their possible synergistic effects, still need to be investigated [33].

Many flavonoids may upregulate the antioxidant Nrf2 pathway and related phase II detoxification enzymes such as SOD and CAT for maintaining brain health during ROS overproduction and chronic neuroinflammation leading to BBB dysfunction and the onset and progression of neurodegenerative disorders. It is interesting to note that natural polyphenols follow the concept of hormesis, a biphasic dose–response process by which small, nontoxic stresses or mild stress are used to induce cellular adaptive responses that protect biological systems against subsequently large and potentially lethal stresses of the same, similar, or different nature. Therefore, polyphenols of *F. benjamina* can be considered as hormetic nutrients as they are capable of inducing health benefits in a dose-dependent manner. In particular, flavonoids can inhibit neurotoxic damage and mitochondrial dysfunction, ultimately enhancing clinical efficacy to therapy. On the other hand, a high dose of natural extracts can be toxic to cells and animal models, leading to the inhibition of cellular antioxidant pathways and the onset and progression of nervous system disorders associated with oxidative stress and chronic inflammation. The field of hormetic/adaptive responses activated by nutritional compounds and/or supplements in enhancing endogenous redox defense systems and inhibiting neuroinflammatory pathways is emerging as promising preventive and therapeutic approach in several chronic disorders associated with oxidative damage. Dose is a crucial determinant for inducing protective or harmful effects and should be carefully evaluated. Therefore, finding the optimal dose to induce anti-neurodegenerative is of crucial importance to achieve protection and enhance clinical efficacy of therapy. Much recent evidence has elucidated the effects of hormetic nutrients at low/moderate concentrations by activating endogenous antioxidant pathways and inhibiting pro-inflammatory pathways in vitro and in vivo dose-dependently. This is in agreement with the results of our study [34,35].

This study attempted to address this knowledge gap by defining the neuroprotective benefits of *F. benjamina* extracts on H_2_O_2_-induced oxidative stress in SH-SY5Y cells and validating these results in an in vivo ONC injury model. We identified the most bioactive fraction of *F. benjamina* extract and systematically assessed its impact on mitochondrial respiration, membrane potential, the expression of apoptosis-related genes, antioxidant enzyme levels, and neurotrophic signaling molecules. This provides new insights into the potential use of *F. benjamina* in neuroprotective strategies. Our results reveal a complex mechanism that goes beyond simple activity for antioxidants.

According the hormesis concept, neuronal dysregulation of the Nrf2 pathway may be an important cause of selective susceptibility under inflammatory conditions [35]. It has been reported that flavonoids may inhibit oxidative stress and neuroinflammation, which is associated to the pathogenesis of various neurological disorders including stroke, Alzheimer’s, Parkinson’s diseases, and so on [36].

### 3.1. Cytoprotective Effects of F. benjamina Leaf Extract

Among the five plant leaf extracts screened, *F. benjamina* exhibited the most pronounced cytoprotective effects against H_2_O_2_-induced cell damage, preserving cell viability at 62–65% at concentrations ranging from 50 to 100 μg/mL. Although 200 μg/mL was included in the initial screening, *F. benjamina* did not show further improvement in cell viability at this dose and exhibited a modest decline relative to 50–100 μg/mL, suggesting the beginning of a concentration-dependent ceiling or early cytotoxic effect. Consequently, subsequent mechanistic experiments focused on concentrations that demonstrated reproducible neuroprotection. A more detailed toxicity curve, including higher concentrations, will be required in future studies to fully establish the therapeutic window.

It is important to note that the 80% fraction had better protective effects, which suggests that this fraction has a unique concentrated composition of bioactive compounds. These findings consistently match with previous studies on Ficus species, which indicate a range of biological activities, including antioxidative, anti-inflammatory, and neuroprotective effects [37].

### 3.2. Mitochondrial Functional Modulation

One of the characteristics of neurodegenerative diseases is impaired mitochondrial activity, which leads to the formation of free radicals and further impairment of mitochondrial function [10]. For this reason, we examined whether the 80% fraction influences mitochondrial activity or protective activity. We observed that exposure to H_2_O_2_ led to pronounced mitochondrial stress, as evidenced by elevated oxygen consumption rates, increased non-mitochondrial respiration, enhanced maximal respiratory capacity, and higher ATP synthesis. *F. benjamina* 80% fraction pre-treatment regulated most of these parameters back to control levels, suggesting the restoration of mitochondrial homeostasis. There were no alterations in proton leak in *F. benjamina* 80%-fraction-treated cells, even though the respiratory indicators had been normalized and the membrane potential remained unchanged (as shown by JC-1 staining). This shows a new mechanism of action. The reported protective effect may be linked to mitochondrial mechanisms that involve proton leak, which mitigates ROS generation while maintaining the potential of the mitochondrial membrane. This indicates that the 80% methanolic fraction of *F. benjamina* extract may facilitate mild mitochondrial uncoupling as a neuroprotective strategy. Mild uncoupling lowers the production of ROS while still allowing enough ATP synthesis to lower oxidative stress without negatively affecting cellular energy processes [38].

### 3.3. Gene Expression Modulation by F. benjamina Extract

Gene expression study showed that the 80% methanolic fraction of *F. benjamina* protects neurons by mechanisms involving multiple molecular pathways, as evidenced by coordinated changes in gene expression and corresponding functional outcomes. Direct testing through pathway inhibition or gene silencing would provide definitive causal confirmation and represents an important direction for future mechanistic studies. Being exposed to oxidative stress caused by H_2_O_2_ caused important antioxidant defense genes like SOD2 and CAT to be downregulated. SOD2 is an essential element of the mitochondrial antioxidant defense system because it turns superoxide radicals into hydrogen peroxide (H_2_O_2_), which is less reactive and is capable of crossing the mitochondrial membrane [39]. The absence of SOD2 activity can lead to various pathological phenotypes in metabolically active tissues, especially in the central nervous system [40]. CAT is an important part of the cell’s defense against H_2_O_2_ buildup because it breaks it down into H_2_O and O_2_ with the help of glutathione reductase [41]. The 80% methanolic fraction of *F. benjamina* successfully restored the expression levels of SOD2 and CAT to levels close to that of the control groups, indicating the reactivation of innate antioxidant defenses. This restoration possibly helps with the neuroprotective effects that have been observed and corresponds with the idea that polyphenols that are extracted from plants activate antioxidant response elements to make cells more efficient at defending against oxidative damage [42,43].

The 80% methanolic fraction of *F. benjamina* significantly regulated expression of apoptotic pathway genes, restoring the levels of anti-apoptotic BCL-2 and BCL-xL without significant effect on pro-apoptotic protein BAX. This shift was reflected in an increased BCL-2/BAX ratio (from 0.43 to 0.63) and decreased BAX/BCL-xL ratio (from 2.5 to 1.67), indicating a change towards a pro-survival state, though with incomplete restoration of homeostatic balance. These findings are consistent with previous reports demonstrating that flavonoids and phenolic compounds can regulate apoptotic protein expression under oxidative stress in neuronal models [42,44,45]. Another significant observation was that the fraction partially restored BDNF expression, which was markedly suppressed by H_2_O_2_-induced oxidative stress (0.35-fold), enhancing it to a moderately improved level (0.55-fold) upon pre-treatment. BDNF upregulation is important for neurodegenerative conditions, as BDNF promotes neuronal survival, differentiation, and synaptic plasticity [46]. AADC expression was not significantly altered by the 80% fraction, though the relevance of neurotransmitter synthesis pathways in this cell culture model is limited and would require validation in a differentiated neuron model. These results are in agreement with earlier research indicating minimal regulation of enzymes associated with neurotransmitter metabolism by plant extracts, regardless of their considerable neuroprotective properties [47]. The collective gene expression data indicates that the *F. benjamina* 80% fraction mediates neuroprotection via a multiple molecular pathways. This approach addresses both the immediate consequences of oxidative damage by enhancing antioxidant defenses and the downstream apoptotic cascades and neurotrophic impairments that add to neuronal death in neurodegenerative disorders, making it a multi-target approach that is increasingly recognized as vital for effective neuroprotection.

We acknowledge that SH-SY5Y cells are a human neuroblastoma cell line rather than primary neurons. However, this cell line is widely used in neuroprotection studies due to several advantages: (1) they express neuronal markers and catecholaminergic characteristics; (2) they provide a reproducible, homogeneous model for high-throughput screening of neuroprotective compounds; (3) their response to oxidative stress (mitochondrial dysfunction, apoptosis, and ROS generation) recapitulates key features of neuronal injury; and (4) they allow mechanistic studies that would be challenging in primary neurons due to heterogeneity and limited lifespan. While SH-SY5Y cells have limitations including transformed phenotype and potential differences from mature neurons, our in vivo validation in the ONC model using retinal ganglion cells—true central nerve system (CNS) neurons—supports the translational relevance of our in vitro findings. Future studies should confirm these effects in primary neuronal cultures or iPSC-derived neurons.

### 3.4. In Vivo Neuroprotection

Optic nerve crush injury is a useful model to study the response of central nervous system neurons to injury and neuroprotection strategies [48,49]. For that reason, we chose to study the neuroprotective effect of the *F. benjamina* 80% fraction in a mouse model. According to current anatomical estimates, the mouse vitreous chamber volume is approximately 5–7 μL [50]. Therefore, a 3 μL intravitreal injection corresponds to roughly 40–60% of the total vitreous volume—lower than earlier assumptions based on outdated eye volume estimates of 4–5 μL. In our laboratory, we have extensive experience with this injection protocol, and when it is performed correctly, with a slow injection rate (1–2 min), accurate needle positioning to avoid lens or retinal contact, and a brief pause to allow pressure equilibration before needle withdrawal, we do not observe any structural or functional retinal damage [51]. Consistent with this, vehicle-injected control eyes in our previous studies exhibited no detectable retinal ganglion cell loss or architectural abnormalities relative to uninjected controls, indicating that the injection procedure itself does not contribute to the measured outcomes. Moreover, a 3 μL injection volume, as used in several recent studies [52,53,54], offers practical advantages by minimizing variability in drug delivery and improving distribution within the vitreous chamber, thereby ensuring more consistent therapeutic exposure in the small mouse eye. The treatment led to increased survival of RGCs, especially when administered immediately following ONC, indicating its potential effectiveness in acute neurodegenerative injury settings. These results align well with neuroprotective studies conducted in other Ficus species such as *Ficus platyphylla* [55] and *Ficus deltoidea* Jack [56].

Intravitreal injection was selected for this proof-of-concept study to maximize retinal bioavailability and establish the therapeutic potential of our flavonoid fraction. Importantly, pathological conditions such as ischemia and optic neuropathy are characterized by blood–retinal barrier (BRB) breakdown and increased vascular permeability, as demonstrated by Rappoport et al. [51]. This compromised barrier in diseased states may facilitate systemic delivery of flavonoids to retinal tissues. Future studies will evaluate oral and intraperitoneal administration routes to assess BRB penetration, systemic bioavailability, and therapeutic efficacy under both healthy and pathological conditions, which is essential for clinical translation.

Our findings propose that the *F. benjamina* 80% fraction exerts a neuroprotective effect involved and activated by several mechanisms including (1) maintenance of mitochondrial membrane potential despite mild uncoupling, (2) normalization of mitochondrial respiration parameters, (3) upregulation of antioxidant enzymes, (4) modulation of apoptotic pathways, and (5) partial restoration of neurotrophic support.

This multi-target strategy may provide advantages over conventional neuroprotective approaches, which have predominantly been unsuccessful in clinical studies for neurodegenerative disorders. *F. benjamina* 80% extract’s potential to change how mitochondria function and boost resistance to oxidative stress is particularly relevant for diseases like Alzheimer’s, Parkinson’s, and other neurodegenerative diseases [57,58,59]. Despite the promising results of this research, deeper investigations into the mechanisms behind neuroprotection are essential. We did not include a vehicle injection control in the ONC model, which would help distinguish specific extract effects from potential injection-related neuroprotection. Enhancing delivery methods may also increase the effectiveness of polyphenols of *F. benjamina*. Future investigations should focus on evaluating the safety, efficacy, correct dosage, method of administration, treatment frequency, and duration.

## 4. Materials and Methods

### 4.1. Cell Line

SH-SY5Y cells were cultured in DMEM (Dulbecco’s Modified Eagle Medium) supplemented with 10% heat-inactivated fetal bovine serum, 1% L-glutamine, and 1% penicillin–streptomycin ((all from Biological Industries, Kibbutz Beit Haemek, Israel) and incubated at 37 °C with 5% CO_2_.

### 4.2. Treatment of Plant Material

Five different plants were used in the study: *F. benjamina* L., voucher specimen number (# 10 99 54); *Phlomis viscosa* Poir., voucher specimen number (# 10 11 76); *Urtica dioica* L., voucher specimen number (# 10 54 28); *Anchusa strigosa* [Soland.], voucher specimen number (# 10 29 71); and *Varthemia iphionoides* Boiss. & C.I.Blanche, voucher specimen number (# 10 25 32). All plants were deposited at the Herbarium at the National Natural History Collection of the Hebrew University of Jerusalem. The plants were sourced from a greenhouse at Ben-Gurion University of the Negev (Beer-Sheva, Israel). Ethanolic extracts were prepared from dried leaf material. Before extraction, fresh leaf material was shade-dried, finely ground, and soaked in 96% ethanol for 48 h in an orbital shaker incubator (120 rpm) at 25 °C. The mixture was then centrifuged at 2000 rpm for 10 min (Beckman Coulter, Brea, CA, USA). After centrifugation, the supernatant was collected and evaporated using a lyophilizer (Merck, Hamburg, Germany). The resulting pellet was weighed and dissolved to the desired concentration.

The pellet was dissolved in a minimal amount of 96% ethanol and diluted with medium to the necessary concentrations for experiments.

### 4.3. Identification of Plant Compounds

The extract was fractionated with rising methanol gradient: 0%, 20%, 40%, 60%, 80%, and 100%, as previously described [26]. The methanol fractions were evaporated. Identification of phytochemicals was carried out in the fraction by high-performance liquid chromatography (HPLC, Dionex Softron GmbH, Germering, Germany), liquid chromatography–electrospray ionization–mass spectrometry (LC-ESI-MS, Agilent 1100LC series, Waldbronn, Bremen, Germany), and matrix-assisted laser desorption/ionization–time-of-flight mass spectrometry (MALDI-TOF-MS, Bruker Daltonics, Bremen, Germany). The chromatographic conditions were described previously [26]. Briefly, the separation was performed using a Betasil C-18 column, 5 μm, 250 × 0.46 mm (Thermo-Hypersil, Paisley, UK). The mobile phase was solution A, consisting of water and acetic acid (97:3 *v*/*v*), and solution B, which was methanol. The injection volume was 20 µL, and the mobile phase flow rate was 1.0 mL/min; the UV detector was set at a wavelength of 360 nm.

### 4.4. Cell Viability Assay

SH-SY5Y cells were seeded at 1 × 10^4^ cells/well, in a final volume of 100 µL, in 96-well flat-bottomed microtiter plates. After 24 h of incubation, cells were treated with H_2_O_2_ (50–300 µM) for 12 h to determine a suitable concentration for oxidative stress induction. H_2_O_2_ was freshly prepared from 30% stock solution prior to each experiment.

For neuroprotection treatment, cells were pre-treated with plant extract or fraction for 6 h at a range of concentrations (25, 50, 100, and 200 µg/mL). Subsequently, H_2_O_2_ (250 μm) was added to the media, and the cells were incubated for an additional 12 h. At the end of incubation, 50 μL of XTT solution (Biological Industries, Kibbutz Beit Haemek, Israel) was added to each well, and the plates were incubated at 37 °C for 1 h. Absorbance was measured at 450 nm against a reference wavelength of 650 nm using a microplate reader (TECAN plate reader, Männedorf, Switzerland).

### 4.5. JC-1 Mitochondrial Membrane Potential Assay

SH-SY5Y cells were seeded at the concentration of 5 × 10^4^ cells/well in the DMEM growth medium and incubated in 6-well plates at 37 °C under 5% CO_2_. The experimental method was described in Section 2.3. The cells were collected with 0.25% trypsin/EDTA solution and washed with PBS twice at 1500 rpm for 5 min. The cells were re-suspended in JC-10 working solution (Abcam, Cambridge, UK) and incubated for 30 min before being washed again and analyzed by flow cytometry.

### 4.6. RNA Isolation and Assessment of mRNA Expression Using Real-Time RT-PCR

#### 4.6.1. RNA Extraction

SH-SY5Y cells were pre-treated with 50 µg/well with the 80% fraction for 6 h, followed by the addition of H_2_O_2_ for another 12 h. RNA extraction was performed using the GENEZOL TRIRNA PURE KIT (Geneaid, New Taipei, Taiwan) according to the manufacturer’s protocol. The concentration of extracted RNA was estimated by optical density measurement (A260/A280 ratio) with a NanoDrop 1000 Spectrophotometer (Wilmington, DE, USA).

#### 4.6.2. cDNA Synthesis

The cDNA synthesis reaction mixture was composed of 1 μg of extracted RNA, 1 μg of random hexonucleotides primers and 1 μM deoxyribonucleotides (dNTP), 10 units of RNase inhibitor, and Moloney Murine Leukemia Virus SuperScript II^®^ Reverse Transcriptase (M-MLV RT, Invitrogen, Modi’in, Israel). Then, the reaction tubes were maintained at 37 °C for 2 h and heat-inactivated at 85 °C for 5 min.

#### 4.6.3. Real-Time PCR

Real-time PCR was carried out using SYBR™ Green Universal Master Mix (Thermo, Modi’in, Israel). The QuantStudio 1 System (Thermo Fisher Inc., Modi’in, Israel) was used for performing all amplification reactions in a total volume of 20 µL. Each well in this experiment contained 1 µL of cDNA, 70–100 nM of each primer (Sigma Aldrich, Rehovot, Israel), and 12.5 µL of SYBR green PCR Master Mix. The applied primer sequences for quantitative PCR are presented in Table 2.

### 4.7. Evaluation of Mitochondrial Respiration and Cellular Bioenergetics

SH-SY5Y cells were seeded at 5 × 104 cells/well in XF8 cell culture plates. After 24 h of incubation, the cells were treated with the *F. benjamina* 80% fraction for 12 h followed by H_2_O_2_ induction as described above. Prior to assay, the growth medium was exchanged with 200 μL of XF assay medium-modified DMEM and incubated at 37 °C without CO_2_ for 1 h. The cellular oxygen consumption rate (OCR) was measured using the Seahorse XF HS Mini Analyzer and software (Seahorse_Wave_Desktop_V2.6, Seahorse Bioscience, Agilent, Santa Clara, CA, USA). Basal OCR was measured before sequential injection of the following inhibitors: oligomycin 1.5 µM, carbonyl cyanide-4-(trifluoromethoxy) phenylhydrazone (FCCP) 2 µM, and rotenone + antimycin 0.5 µM, to measure minimal, maximal, and non-mitochondrial respiration, respectively. The data was exported to Excel and normalized to cell density in each well, quantified by cell counting of each well.

### 4.8. Establishment of Optic Nerve Crush Model and Intravitreal Injections

#### 4.8.1. Animals

Male C57BL/6 mice (8 weeks old) were purchased from Envigo RMS, Jerusalem, Israel. Animals were fed with standard food and water in a 12 h light/dark cycle. The mice were maintained and handled in accordance with the ARVO Statement for the Use of Animals in Ophthalmic and Vision Research and the National Institute of Health guidelines. The protocols of animal experiments were approved by the Institutional Animal Care Committee at Rabin Medical Center, Israel (authorization number: 010521).

#### 4.8.2. Optic Nerve Crush (ONC) Model

The induction of ONC has been previously described [60]. In brief, C57BL/6 mice were anesthetized with intraperitoneal injection of ketamine (80 mg/kg) (Fort Dodge Laboratories, Fort Dodge, IA, USA) and xylazine (4 mg/kg) (VMD, Westlands, Kenya). The right optic nerve was crushed by applying forceps at a point 2.5–3.0 mm posterior to the globe for 7 s, three times consecutively. Intravitreal injection of the 80% fraction (3 μL) was performed using a using a 33-gauge Hamilton needle (Hamilton, Reno, NV, USA), entering through the superotemporal sclera and delivering the solution slowly to avoid acute intraocular pressure elevation or mechanical disruption of the retina 1h before crush or immediately after. The contralateral (left) uninjured eye was served as a control.

### 4.9. Statistical Analysis

All statistical analyses and graphs were performed with GraphPad Prism 10 (GraphPad Software, La Jolla, CA, USA). The results of the study were expressed as mean ± SD. Data was analyzed by using a one-way ANOVA followed by Tukey’s HSD post hoc statistical test, or two-way analysis of variance test (ANOVA) followed by Bonferroni post hoc for multiple comparisons. Values with *p* < 0.05 were considered significant.

## 5. Conclusions

Our findings provide new pharmacological validation for the ethnomedicinal use of Ficus species. Within TCM, related Ficus plants are applied to treat inflammatory conditions, promote circulation, and support recovery from weakness concepts that parallel our observed antioxidant, mitochondrial-stabilizing, and pro-survival effects. Notably, this study is the first evidence that the 80% methanolic fraction of *F. benjamina* directly confers neuroprotective effects at both the cellular and mitochondrial levels. Traditional records have indicated extensive restorative properties; however, our findings provide mechanistic evidence that the flavonoid-rich fraction influences oxidative stress pathways, apoptosis, and neurotrophic signaling.

To our knowledge, this study provides the first mechanistic validation of *F. benjamina*’s neuroprotective effectiveness, thereby extending its ethnopharmacological significance into the neurological realm. This makes the extract a good prospect for further development in integrative medicine, which combines ideas from traditional medicine with modern molecular evidence.

## Figures and Tables

**Table 1:** Phytochemicals identified in the 80% fraction of *F. benjamina*.

Compound	Molecular Structure	Methods of Identification	Conc., mg/kg
Caffeic acid	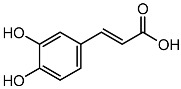	HPLC LC-ESI-MS	7.6 ± 0.58
Quercetin 3-*O*-rutinoside	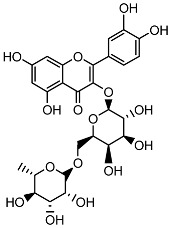	HPLC LC-ESI-MS	11.5 ± 0.21
Kaempferol 3-*O*-robinobioside	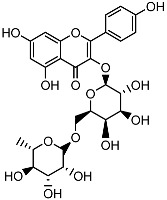	HPLC LC-ESI-MS	4.3 ± 0.19
Kaempferol 3-*O*-rutinoside	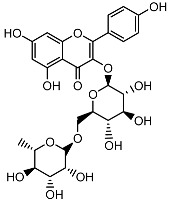	HPLC LC-ESI-MS	3.9 ± 0.55

**Table 2:** Primer sequences for quantitative PCR.

Gene	Primer Pair	Sequence (5′ → 3′)
* BDNF *	FP RP	ATGACCATCCTTTTTCCTTACT GCCACCTTGTCCTCGGAT
* AADC *	FP RP	AACAAAGTGAATGAAGCTCTTC GCTCTTTGATGTGTTCCCAG
* SOD2 *	FP RP	AGGCCGTGTGCGTGCTGAAG CACCTTTGCCCAAGTCATCTGC
* CAT *	FP RP	CCTTTCTGTTGAAGATGCGGCG GGCGGTGAGTGTCAGGATAG
* Bcl-2 *	FP RP	GATTGAGGGATCGTTGCCTTA CCTTGGCATGAGATGCAGGA
* BAX *	FP RP	GCGAGTGTCTCAAGCGCATC CCAGTTGAAGTTGCCGTCAGAA
* Bcl-xl *	FP RP	CTTTGCCTAAGGCGGATTTGAA AATAGGGATGGGCTCAACCAGTC

## Data Availability

The original contributions presented in this study are included in the article/Supplementary Materials. Further inquiries can be directed to the corresponding authors.

## Supplementary Materials

The following supporting information can be downloaded at: https://www.mdpi.com/article/10.3390/ijms262311746/s1.

## Abbreviations

AADC	Aromatic L-amino Acid Decarboxylase
BAX	BCL2-associated X
BCL2	B-cell lymphoma 2
BDNF	Brain-Derived Neurotrophic Factor
CAT	Catalase
CNS	Central nervous system
DMEM	Dulbecco’s Modified Eagle Medium
FCCP	Carbonyl cyanide-4-(trifluoromethoxy) phenylhydrazone
HPLC	High-performance liquid chromatography
LC-ESI-MS	Liquid chromatography–electrospray ionization–mass spectrometry
MALDI-TOF-MS	Matrix-assisted laser desorption/ionization–time-of-flight mass spectrometry
OCR	Oxygen consumption rate
ONC	Optic nerve crush
SOD2	Superoxide Dismutase 2
RGC	Retinal ganglion cell
ROS	Reactive oxygen species
