# Supplementary material for: Multi-Target Neuroprotective Effects of Flavonoid-Rich Ficus benjamina L. Leaf Extracts: Mitochondrial Modulation, Antioxidant Defense, and Retinal Ganglion Cell Survival In Vivo
Source: Int J Mol Sci. 2025 Dec 4;26(23):11746. doi: 10.3390/ijms262311746 (PMC12692513; doi:10.3390/ijms262311746)
Supplement: Supplementary file 1 [file ijms-26-11746-s001.zip › ijms-3967522-supplementary.pdf]

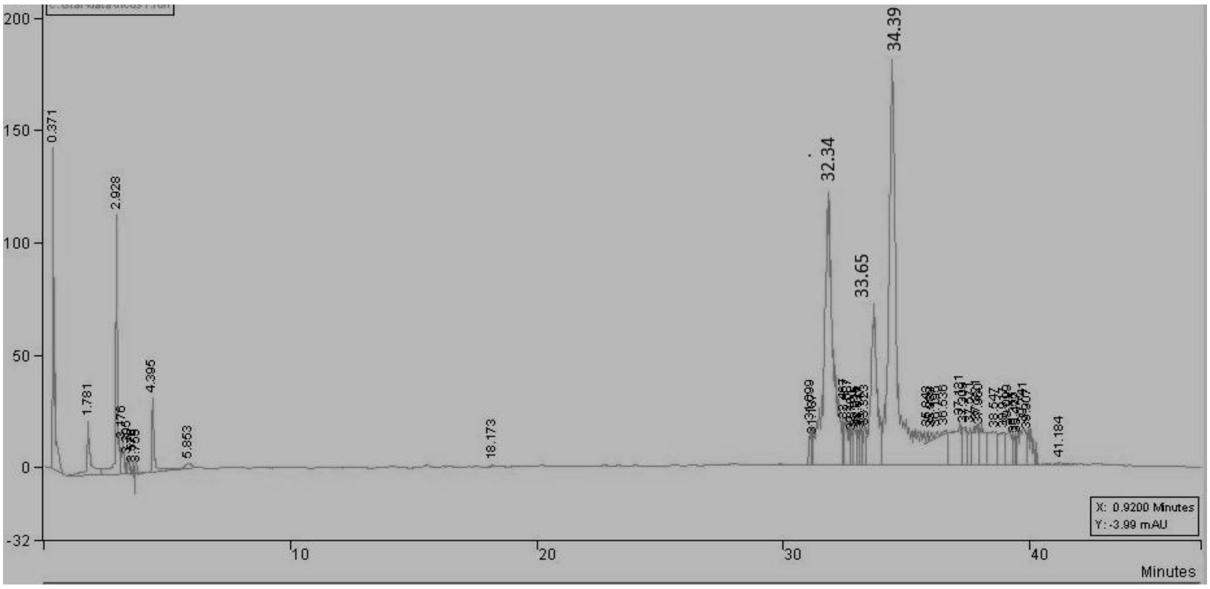

Supplementary Figure S1. HPLC chromatograms of flavonoids in the extract of *F. benjamina.* The flavonoid fraction (80%-MeOH) was analyzed by HPLC. Gradient elution was performed with solution A, composed of water-acetic acid (97 : 3 V/V) and solution B – methanol. Leaf ethanol extracts of *F. benjamina* were subjected to repeated purification and HPLC for flavonoid collection for further identification.
